# Tobacco Use and Associated Factors in Patients Presenting to a Psychiatric Emergency Room

**DOI:** 10.1155/2018/8102165

**Published:** 2018-06-13

**Authors:** Ashely Collins, Oluwadamilare Ajayi, Savannah Diamond, William Diamond, Suzanne Holroyd

**Affiliations:** ^1^Department of Psychiatry and Behavioral Medicine, Marshall University Joan C. Edwards School of Medicine, Huntington, WV 25701, USA; ^2^Department of Psychiatry and Neurobehavioral Science, University of Virginia School of Medicine, Charlottesville, VA 22908, USA

## Abstract

**Background:**

Rates of cigarette smoking among the public and individuals with mental illness have been well documented. Studies have demonstrated that prevalence of smoking among individuals with mental illness remains elevated compared to the general population and as a distinct subgroup, individuals with mental illness consume more than a third of cigarettes sold in the U.S. However, information on rates of smoking among patients presenting to a psychiatric emergency room (ER) is lacking. This study assesses this understudied population for smoking prevalence and associated factors.

**Methods:**

A retrospective chart review of 203 distinct psychiatric ER patients was conducted. Demographics, tobacco use, substance use, psychiatric diagnoses, and family history were noted and analyzed with SPSS.

**Result:**

Tobacco use rates were noted to be nearly fifty percent and significant associations were found with active suicidal ideation, alcohol use disorders, illicit drug use disorders, and history of prior psychiatric hospitalization.

**Conclusion:**

Tobacco use among psychiatric ER patients is much higher than that of the general population and associated with active suicidal ideations, alcohol use disorders, and illicit substance use disorders. These findings should be considered in the evaluation and expectant management of these patients by their clinicians and healthcare providers.

## 1. Introduction

Individuals with diagnosed mental disorders and significant psychological distress (increased likelihood of diagnosable mental illness) (psychological distress as assessed using the Kessler 6 scale (K6 scale), a screening tool for mood and/or anxiety disorders and to identify individuals with increased likelihood of having diagnosable mental illness and associated function limitations, for health population surveys) have consistently been shown to have higher cigarette smoking rates when compared to the general population [1–4]. Per a recent morbidity and mortality weekly report (MMWR) by the Centers for Disease Control and Prevention (CDC) on current smoking in U.S. adults, cigarette smoking prevalence is nearly three times greater in individuals with significant psychological distress (40.6%) when compared to those without significant psychological distress (14.0%) and the general population (15.1%) [2]. Prevalence of smoking and intensity of smoking have also been found to increase with an increase in the number of comorbid mental illnesses diagnosed [1, 3]. Additionally, several studies have suggested that individuals with mental illness and/or substance abuse disorders while accounting for less than a quarter of the U.S. adult population consume 30.9%-44% of cigarettes sold in the U.S. [4–6].

Certain disorders most especially schizophrenia, schizoaffective, bipolar, depression, anxiety, and substance abuse disorders have higher rates of tobacco use [6–8]. The prevalence of nicotine dependence ranges from an astonishing 58% to 92% in patients with schizophrenia, 60% to 70% in patients with bipolar disorder, and 31% to 61% in patients with major depression [7, 9]. There are numerous theories that explain why certain populations have such high smoking rates. Nicotine may relieve anhedonia through dopamine release in the mesolimbic reward center; thus smoking may be one of the only activities in which patients with schizophrenia and major depression find pleasure [7]. In schizophrenia, smoking may normalize sensory deficits such as auditory sensory gating and smooth pursuit eye movements by impacting the P50 system. Further, nicotine may help relieve negative symptoms via increasing dopaminergic activity in mesocorticolimbic pathways and prefrontal cortex. Smoking may also decrease adverse effects from antipsychotics [7, 10]. Interestingly, those with schizophrenia and depression attribute greater advantages than disadvantages to smoking when compared to the general population, even though all groups equally recognized the negative effects of smoking [7].

Although there has been a decline in smoking across the United States and its territories [2, 11], the rates of decline among individuals with mental illness have been much less significant (3.2% compared to 31% decline between 2005 and 2015) [2, 3, 12]. Smoking related mortality is high in the mentally ill population as evidenced by one study of 600,000 patients where tobacco related conditions were attributed to 53% of total deaths in schizophrenia, 48% in bipolar disorder, and 50% in major depressive disorder patients [13].

Despite the known data on smoking and its relationship with specific psychiatric diagnoses or psychological distress, it is surprising that there is a paucity of studies examining tobacco use of psychiatric patients in specific treatment settings, such as outpatient, inpatient, consult, or ER. Conceivably, patients seen in different settings may have different rates of smoking as well as specific associated clinical factors and outcomes. A literature review yielded a limited number of studies, and those were focused on the psychiatric inpatient and outpatient populations [14–19]. Further characterization of psychiatric patients who smoke in different treatment settings may allow strategies to address this health issue through tactics best suited to that setting, as well as look for psychiatric correlates to smoking behavior.

To our knowledge, there has been no study of tobacco use in patients who present to a psychiatric emergency room (ER). This is an important group to study given the acuity of presentation in this population. In this study, we examined the prevalence of cigarette smoking and associated factors among those patients presenting to a psychiatric ER.

## 2. Methods

In our study, the records of 226 distinct consecutive psychiatric ER patients who visited an academic medical center in Virginia over a two-month period were reviewed retrospectively. Smoking prevalence was examined for these patients, with 23 subjects being excluded due to incomplete tobacco use data. Based on patient report during standard psychiatric interview, medical records review, and family/caregiver report where appropriate, data was compiled on our sample of 203 patients. These data included information on their tobacco/cigarette use, alcohol use and alcohol intoxication status at ER visit, family history of alcohol abuse, substance use, and the type of substance used (such as cocaine, marijuana/THC, opioids, and other illicit substances). Additionally, data on suicidal ideations, homicidal ideations, and psychiatric and neurologic history of the patients as well as their families were also assessed. Lastly, demographic data on age, gender, race, and marital and employment status were noted.

A patient was considered a tobacco user, if they responded in the affirmative to enquiry about current cigarette smoking. Alcohol intoxication status of the patient was confirmed by reviewing records on blood alcohol levels at the time of visit. Urine drug screen (UDS) data was unavailable.

The data gathered was organized and entered into SPSS Ver. 22 for analysis. Data was analyzed with Fisher's exact test, with statistical significance assumed at a level P ≤ 0.05. This study was approved by the institutional IRB.

## 3. Results

Of the 203 patients, 101 (49.8%) used tobacco. Of those 101 tobacco users, 48 (47.5%) were admitted to an inpatient service. The mean age of the sample was 41.2 years. More demographic information is summarized in Table 1. No significant differences were found between ER patients who smoked versus those who did not in the domains of age, gender, race, marital status, employment, or living situation.

Active suicidal ideations (p<0.01) and illicit substances use (p<0.01) were found to have statistically significant associations with cigarette smoking. Further analysis revealed this observation held for cocaine (p<0.01), THC (p<0.01), and other illicit substances (p< 0.05). Additionally, alcohol use disorders (p<0.001) and history of prior psychiatric hospitalizations (p<0.05) had significant associations with cigarette smoking (Table 2). Family history of alcoholism was reported in 35.6% of smokers in this sample and was highly associated with tobacco use (p<0.01).

Interestingly, our data did not yield any statistically significant associations between tobacco use and specific psychiatric diagnoses. Some notable examples are major depressive disorder (p = 0.886) and bipolar affective disorder (p = 0.208). Statistically significant associations were not found with family histories of substance use (p = 0.444) and tobacco use (p = 0.302).

Because this was an exploratory study examining factors associated with tobacco use in the psychiatric ER, an understudied environment, a Bonferroni correction was not made.

## 4. Discussion

The psychiatric ER treatment setting represents a population of patients that has been understudied. Our study had several notable findings including the high prevalence of tobacco use, the strong association between smoking and active suicidal ideation, and the strong association to other substance use disorders (illicit and alcohol).

In this study, the prevalence of tobacco use among psychiatric ER patients was much higher compared to the general population (49.8% versus 15.1%) but comparable to the inpatient psychiatric population (42-57.3%) [15–17]. The rate however was less than that among psychiatric outpatients (52-61%) [18, 19]. There were other interesting findings comparing the psychiatric ER population to inpatient and outpatient focused studies. A study of psychiatric inpatients found a significant association between cigarette smoking and a history of suicide attempts while our study of psychiatric ER patients did not [16]. Additionally, a study in the outpatient setting found no association of smoking to alcohol use disorders while in the psychiatric ER setting, there was a very high association of tobacco use and alcohol use disorders [18]. This shows that some notable variations exist among psychiatric patients/populations in a variety of different treatment settings.

Strong associations were found between tobacco use and illicit substance use. Further, strong associations were found between tobacco use and active suicidal ideations as well as a history of prior psychiatric admissions. These observations indicate that the psychiatric ER patients who smoke may be a more psychiatrically ill group. This study may provide useful knowledge for those working in a psychiatric ER setting. Presence of smoking may help identify those at risk for alcohol/illicit substance use disorders as well as suicidal ideation.

Surprisingly, our findings did not demonstrate a statistically significant association between tobacco use and diagnoses such as bipolar disorder and major depression that has been demonstrated in previous studies [1, 5, 6, 8, 13]. We consider that the associations noted in the wider population may not exist in the ER patient population. We could not accurately assess any potential association with schizophrenia due to the small number of persons with this diagnosis in this sample (n = 2). Finally, the higher prevalence of tobacco use in psychiatric ER patients suggests an opportunity to begin exploring smoking cessation with these patients. One study showed that individuals receiving mental health treatment are not only less likely to smoke than those with mental health disorders not receiving treatment, but they are more likely to quit smoking even after adjustment for other factors including sociodemographic variables [12]. Developing educational strategies, referral, and resource connection services on smoking cessation that are suited to the acute setting of the psychiatric ER can help ensure that these patients are provided with the necessary support systems to enable them to quit smoking.

Limitations of this study include its sample size, short patient recruitment period, inability to review urine drug screen (UDS) information, reliance on patient report, and retrospective nature. First, while our sample size is adequate given the exploratory nature of this study, a larger sample size would enhance the generalizability of this study to the wider psychiatric ER population. Similarly, the short patient recruitment period adds to this limitation. These could be addressed in future studies by expanding the sampling period and recruiting multiple centers for a larger and broader sampling of the psychiatric ER population. Lastly, as UDS information was unavailable to be reviewed, a reliance on patient report enhances the response bias on the part of the patient. Finally, the retrospective nature of the study carries limitations in that specific variables are assessed from chart review rather than being prospectively examined. This may have affected the results; for example, it is likely that prevalence of illicit substance use may have been higher than reported.

Further research examining the relationship between tobacco use and mental health disorders is warranted. Our findings add to the characterization of tobacco use in psychiatric patients presenting to ER environments.

## 5. Conclusions

Patients in the psychiatric ER have higher rates of tobacco use/cigarette smoking. Additionally, significant associations with active suicidal ideations, substance, and alcohol use are noted in this patient population. These findings should be considered in the evaluation and expectant management of psychiatric patients in the ER setting by their clinicians and healthcare providers.

## Figures and Tables

**Table 1:** Demographic breakdown of sample.

	Tobacco user (%)	Nontobacco user (%)
*Sex*		
Male	55.4	49
Female	44.6	51
*Marital status*		
Single	59.3	51.2
Married	25.6	34.5
Divorced	15.1	11.9
Separated^*∗*^	-	2.4
*Race*		
Caucasian	74.7	79.5
African American	23	15.7
Other	2.3	4.8
*Employment*		
Employed	19.4	28.9
Unemployed	80.6	71.1

^*∗*^-Described themselves as separated but not divorced.

**Table 2:** Observations on tobacco users within sample.

	Tobacco users	Nontobacco users	P-value
	n (%)	n (%)	
Illicit substances	51 (50.5)	12 (11.8)	0.000
(i) Cocaine	18 (17.8)	4 (3.9)	0.003
(ii) Opioids	7 (6.9)	2 (2.0)	0.170
(iii) Stimulants	6 (5.9)	1 (1.0)	0.119
(iv) THC	37 (36.6)	8 (7.8)	0.000
(v) Other illicit substances	9 (8.9)	1 (1.0)	0.019
Any Alcohol use	66 (65.4)	22 (21.6)	0.000
(i) Alcohol use disorder	28 (27.7)	8 (7.8)	0.000
(ii) Alcohol intoxication	17 (16.8)	5 (4.9)	0.007
(iii) Family history of alcoholism	36 (35.6)	18 (17.7)	0.006
Suicidal ideations	41 (40.6)	28 (27.5)	0.055
(i) Active suicidal ideations	24 (23.8)	10 (9.8)	0.009
(ii) Passive suicidal ideations	23 (22.8)	17 (16.7)	0.294
(iii) Current attempt	10 (9.9)	8 (7.8)	0.631
(iv) Overdose	8 (7.9)	6 (5.9)	0.783
(v) Contracting for safety	67 (66.3)	81 (79.4)	0.041
(vi) Homicidal ideations	8 (7.9)	3 (2.9)	0.214
History of suicide attempts	43 (42.6)	31 (30.4)	0.08
Prior psychiatric hospitalization	66 (65.4)	51 (50)	0.023

Contracting for safety: with this status patient would inform care provider if they were experiencing any thoughts of self-harm.

## Data Availability

Data is available on request from the corresponding author.
